# Pseudotumor Phenotype of Sarcoidosis

**DOI:** 10.7759/cureus.80245

**Published:** 2025-03-08

**Authors:** Mohamed Lakhal, Meriem Rhazari, Sara Gartini, Mohammed Meziane, Afaf Thouil, Amal Bennani, Hatim Kouismi

**Affiliations:** 1 Department of Respiratory Diseases, Mohamed VI University Hospital Center of Oujda, Faculty of Medicine and Pharmacy of Oujda, Oujda, MAR; 2 Department of Pulmonology, Research and Medical Sciences Laboratory, Mohamed VI University Hospital Center of Oujda, Faculty of Medicine and Pharmacy of Oujda, Oujda, MAR; 3 Department of Pathological Anatomy and Cytology, Mohamed VI University Hospital Center of Oujda, Faculty of Medicine and Pharmacy of Oujda, Oujda, MAR

**Keywords:** lung process, mediastinal adenopathy, non-caseous granulomas, pseudotumor, sarcoidosis

## Abstract

Sarcoidosis is a benign multi-system granulomatous disease of unknown etiology. It can occasionally present with a pseudo-tumoral appearance, constituting a diagnostic pitfall that should be considered. We report the case of an 84-year-old patient with no significant medical history, admitted for the management of chronic dyspnea associated with chest pain evolving over the past six months, in the context of a preserved general condition. A chest CT scan revealed a pulmonary process, and the histopathological examination confirmed sarcoidosis. Through this case, we highlight the unique presentation of pulmonary sarcoidosis with a pseudo-tumoral pattern, which is often challenging to diagnose. The discrepancy between the subtle clinical presentation and the extent of radiological lesions should, however, raise suspicion of this diagnosis. Histological confirmation is necessary to rule out other potential etiologies, particularly tumors.

## Introduction

Sarcoidosis is a non-necrotizing systemic granulomatosis of unknown origin, whose preferred sites of involvement are the lungs and mediastinum. However, certain phenotypes can be particularly confusing, notably the pseudotumoral form, which, although rare, may be isolated or accompanied by mediastinal lymph node involvement. This form is often confused with various respiratory pathologies, and frequently leads to a delay in diagnosis [1]. It is therefore essential to consider it in the diagnostic evaluation of certain mediastino-pulmonary lesions [2]. Here, we present the case of a patient with pseudotumoral sarcoidosis.

## Case presentation

**Figure 1:**
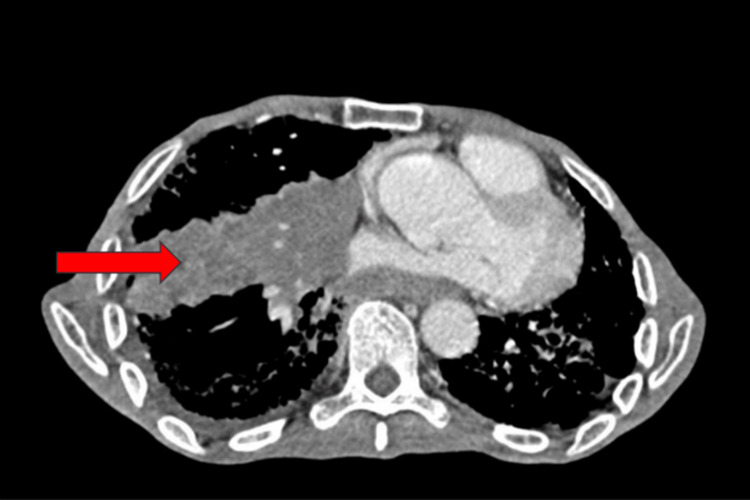
Thoracic CT scan with mediastinal window showing the presence of a right mediastinal-pulmonary lesion. Red arrow: Mediastinal-pulmonary lesion in the middle lobe, poorly defined, containing calcifications, measuring approximately 126 x 82 mm, extending over 200 mm.

This is the case of an 84-year-old patient with no significant past medical history, in particular no known neoplasia. She was admitted for the etiological diagnosis of chronic dyspnea associated with dry cough and chest pain, which had been evolving for six months, in a context of preservation of her general condition. Clinical examination revealed a decrease in vesicular murmurs in the right lung. The rest of the clinical examination was unremarkable. In view of this, the patient underwent a thoracic CT scan (Figure 1), which revealed a mediastino-pulmonary lesional process in the middle lobar region, poorly limited and containing calcifications, measuring approximately 126x82 mm and extending over 200 mm. Multiple bilateral mediastinohilar adenopathies with calcifications were associated.

As part of the etiological workup, the complete blood count (CBC), electrolyte panel, 24-hour proteinuria, and renal function tests were all normal. However, the angiotensin-converting enzyme (ACE) level was elevated. Tumor marker assays were negative, including ACE. The remainder of the laboratory workup showed a negative result for antinuclear antibodies (ANA), antineutrophil cytoplasmic antibodies (ANCA), rheumatoid factor (RF), and anti-cyclic citrullinated peptide (anti-CCP) antibodies (Table 1). 

**Table 1:** Values obtained from patient in comparison with normal laboratory values.

	Our patient	Normal laboratory values
Calcemia	2.40 mmol/L	2.20-2.60 mmol/
Calciuria	8 mmol/L	2.5-7.5 mmol/L
Angiotensin-converting enzyme	80 UI/L	20 – 70 UI/L
Carcinoembryonic antigen	0.3 µg/L	Below 2.5 µg/L
Rheumatoid factors	5 UI/ml	Below 20 UI/ml
Anticyclic citrullinated peptide (anti-CCP)	2 UI/ml	Below 20 UI/ml

**Figure 2:**
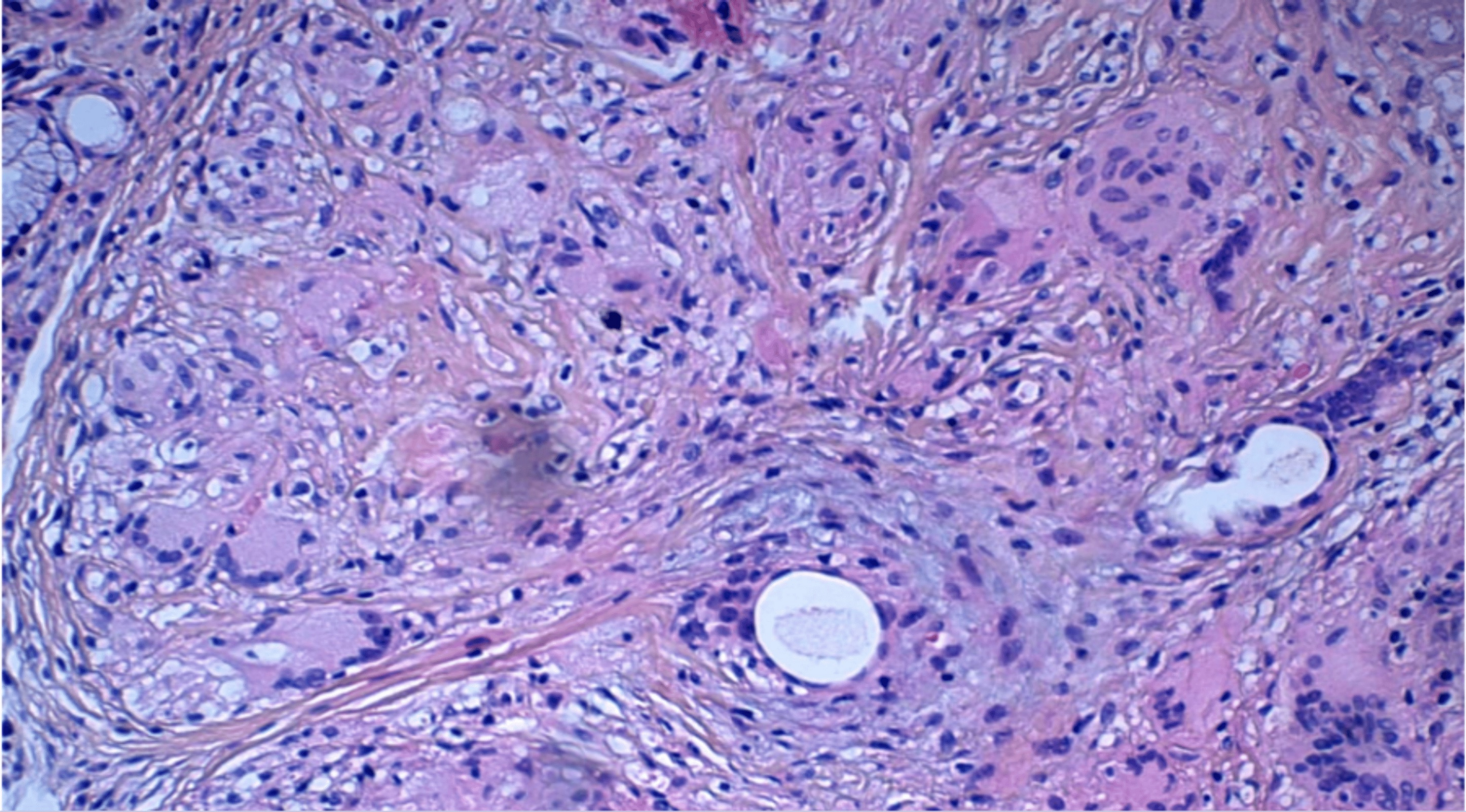
The bronchial mucosa shows multiple epithelioid and giant cell granulomas of varying sizes, non-confluent. No true caseous necrosis is observed.

To complete the etiological investigation, the patient underwent bronchial fibroscopy, which showed extrinsic compression with no visible tumour lesion. Bronchoalveolar lavage revealed no particularities. At this stage, a transthoracic CT-guided biopsy of the pulmonary process was required, the results showed the presence of multiple epithelioid and giant cell granulomas of variable size, non-confluent, without caseous necrosis, primarily suggestive of sarcoidosis (Figure 2).

As part of the work-up, plethysmography showed a total lung capacity of 65%, indicating a moderate restrictive ventilatory disorder. The patient was treated with 40 mg/d of corticosteroids, with potassium supplementation and gastric protection.

## Discussion

Sarcoidosis is a systemic disease of unknown origin, characterized histologically by the presence of non-caseating granulomas. It occurs worldwide, with an estimated prevalence of between 20 and 60 cases per 100,000 people [3]. Mediastino-pulmonary involvement is the most common and almost systematic manifestation. However, certain clinical forms can be complex, presenting with features similar to those of other pathologies. Pseudotumoral lung lesions are rare, but should be considered as part of the diagnosis of pseudoalveolar and nodular lung lesions. Their frequency varies from study to study, ranging from 6% to 34% [4].

According to the two cases of pseudotumoral sarcoidosis reported by Bouhamdi et al., pseudotumoral lesions are pathogenetically the result of the marked confluence of granulomas, which exert pressure on the surrounding alveoli, leading to a pseudoalveolar Heitzman syndrome [5]. They may also result from inflammatory bronchoalveolar infiltration [4].

The clinical features of pseudotumor sarcoidosis are indistinguishable from those of classic forms of the disease, which are generally characterized by clinical latency. However, symptomatic forms are frequently associated with persistent cough, progressive exertional dyspnea, wheezing and sibilant rales [5].

On chest x-ray, sarcoidosis typically presents with peribronchial, subpleural and perilobular septal micronodules, as well as peribronchovascular thickening. This presentation is frequent and generally easy to diagnose [6]. However, lung involvement can sometimes take on an atypical appearance, including nodular or alveolar forms, which may be suggestive of organized pneumonia or malignant pathology [7]. These forms are often accompanied by a marked clinical picture, but their prognosis remains favorable. Opacities are frequently localized in the lung bases. Cavitary and cystic lesions occur in 2-5% of cases, according to a study by Hours et al. [7].

Bronchoscopy is used to explore the bronchial tree and take samples of the bronchial mucosa, transbronchial lung or lymph node biopsies, and bronchoalveolar lavage (BAL). Macroscopic nodular bronchial lesions can be visualized in 10-20% of cases, and stenosing lesions localized in the proximal airways in 1-2% of cases [8]. BAL, when targeted to the affected areas, is particularly useful in eliminating other potential causes of chronic alveolar syndrome, such as eosinophilic lung, and generally reveals a cell profile similar to that seen in classical forms of sarcoidosis. In our patient's case, BAL did not yield conclusive results. Ultrasound-guided transbronchial cytoaspiration proves particularly useful in the presence of mediastinal or hilar lymphadenopathy [8].

The differential diagnosis of pseudotumor sarcoidosis includes several other etiologies, such as lymphoma, bronchioloalveolar carcinoma, and pulmonary fungal infections. Other less common pathologies, such as alveolar proteinosis, oily or drug-induced pneumonia, alveolar metastases of pancreatic cancer, and certain pulmonary vasculitides or connectivitides, should also be considered and ruled out. Berylliosis can be easily excluded in the absence of a suggestive occupational context, while eosinophilic pneumonia and cryptogenic organized pneumonia are much rarer [9].

Regression of sarcoidosis has been observed even without treatment [10]. The efficacy of standard-dose corticosteroid therapy is well established. In a study by Brauner et al., involving a series of twenty patients with sarcoidosis, five of whom had pseudotumor forms, all showed significant improvement on oral corticosteroids [11]. A study by Battesti et al., which followed 746 patients with sarcoidosis, showed that of the 22 patients with pseudotumoral sarcoidosis, 15 required systemic corticosteroid therapy for two years, with a favourable outcome in all cases [10]. In the other six untreated patients, the evolution was spontaneously favorable, with a time to improvement ranging from three to nine months. Only one patient, for whom corticosteroid therapy was contraindicated, developed pulmonary fibrosis [10].

## Conclusions

Pulmonary sarcoidosis in its pseudotumoral form is rare and can be quite confusing, posing a diagnostic challenge that should not be overlooked in respiratory pathology. Histological evidence is necessary to rule out other etiologies, particularly tumors. A discrepancy between the extent of radiological lesions and the subtlety of the clinical picture should alert the clinician. As the disease typically follows a spontaneous favorable course, corticosteroids should only be prescribed in cases of significant respiratory impairment and/or associated visceral damage.
